# Evaluation of Approaches to Identify the Targets of Cellular Immunity on a Proteome-Wide Scale

**DOI:** 10.1371/journal.pone.0027666

**Published:** 2011-11-11

**Authors:** Fernanda C. Cardoso, Joanne S. Roddick, Penny Groves, Denise L. Doolan

**Affiliations:** Queensland Institute of Medical Research, Brisbane, Queensland, Australia; World Health Organization, Switzerland

## Abstract

**Background:**

Vaccine development against malaria and other complex diseases remains a challenge for the scientific community. The recent elucidation of the genome, proteome and transcriptome of many of these complex pathogens provides the basis for rational vaccine design by identifying, on a proteome-wide scale, novel target antigens that are recognized by T cells and antibodies from exposed individuals. However, there is currently no algorithm to effectively identify important target antigens from genome sequence data; this is especially challenging for T cell targets. Furthermore, for some of these pathogens, such as *Plasmodium*, protein expression using conventional platforms has been problematic but cell-free *in vitro* transcription translation (IVTT) strategies have recently proved successful. Herein, we report a novel approach for proteome-wide scale identification of the antigenic targets of T cell responses using IVTT products.

**Principal Findings:**

We conducted a series of *in vitro* and *in vivo* experiments using IVTT proteins either unpurified, absorbed to carboxylated polybeads, or affinity purified through nickel resin or magnetic beads. *In vitro* studies in humans using CMV, EBV, and Influenza A virus proteins showed antigen-specific cytokine production in ELIspot and Cytometric Bead Array assays with cells stimulated with purified or unpurified IVTT antigens. *In vitro* and *in vivo* studies in mice immunized with the *Plasmodium yoelii* circumsporozoite DNA vaccine with or without IVTT protein boost showed antigen-specific cytokine production using purified IVTT antigens only. Overall, the nickel resin method of IVTT antigen purification proved optimal in both human and murine systems.

**Conclusions:**

This work provides proof of concept for the potential of high-throughput approaches to identify T cell targets of complex parasitic, viral or bacterial pathogens from genomic sequence data, for rational vaccine development against emerging and re-emerging diseases that pose a threat to public health.

## Introduction

The development of high throughput techniques to identify the targets of cellular immunity on a proteome-wide scale will facilitate the development of vaccines against complex diseases. Almost all vaccines currently licensed for human use rely on antibody responses against the target pathogen. None are designed to induce protective T cell response, yet T cell responses are implicated as critical in protection against many pathogens, especially those with an intracellular stage such as the causative agents of malaria, leishmaniasis and Chagas disease [1], [2]. CD4^+^ T cells also play a key role in enhancing the pathogen-specific antibody responses [3]. The elucidation of the genome, proteome and transcriptome of important human pathogens, including the *Plasmodium* parasite, has provided a wealth of data that can potentially be mined to identify, on a proteome-wide scale, novel target antigens recognized by T cells and antibodies. However, how to effectively mine this data has proved challenging. In particular, technologies such as conventional protein expression methodologies which are well established on an individual antigen basis often cannot be translated directly to a whole proteome scale.

An important achievement of the scientific community, therefore, has been the development of technologies that allow the high throughput expression of recombinant proteins. These *In Vitro* Transcription and Translation systems (IVTT) or cell-free systems offer several advantages over traditional cell-based expression methods and are suitable for high throughput strategies due to reduced reaction volumes and process time [4], [5], [6]. Additional advantages include easy modification of reaction conditions for improving production of complex proteins, decreased sensitivity to product toxicity, and high yield. Importantly, cell-free systems have proved capable of generating proteins from complex parasites that have been difficult to produce in traditional cell-based systems, such as *Plasmodium* proteins [7], [8], [9], [10]. The most efficient expression to date has been achieved with an *E. coli* cell-free system which has yielded more than 93% efficiency of expression with a panel of 250 *P. falciparum* (*Pf*) proteins [11]. Eukaryotic based cell-free systems are also available with wheat germ, rabbit reticulocytes and insect cells. The eukaryotic lysate is considered by some to provide a better platform for production of complex proteins particularly with regard to post-translational modifications. Recently, up to 75% efficiency of production of *Pf* proteins in the wheat germ system has been reported [12], [13] but this is still less efficient than that observed with the *E. coli* system [11].

The combination of these tools with large-scale cloning strategies, such as recombinatorial cloning, allow the generation of complete proteomes *in vitro* for multiple purposes. Several reports have shown the application of these tools for identifying antibody targets of complex diseases using protein arrays [11], [14], [15]. Such studies have established the feasibility of identifying, from a set of thousands of antigens, the most immunogenic targets of antibody responses which may correlate with protection as indicated by clinical disease stage classification [16] or virus neutralising activity [17].

More challenging is the high throughput elucidation of T cell targets which is clearly of importance for those diseases where cell mediated immunity is implicated in protection, as well as for antibody mediated immunity where T cell help would be beneficial. Recently, the use of the *E. coli* based cell-free system (Rapid Translational system, RTS) for profiling of CD4^+^ T cell responses to vaccinia virus in humans was reported [18]. In that study, 180 predicted open reading frames of the vaccinia genome were expressed in the RTS system and unpurified RTS reaction products were tested for recognition by vaccinia virus-enriched T cell lines derived from 11 Dryvax smallpox vaccines, using ^3^H-thymidine proliferation assays. Another study reported the use of IVTT products affinity purified on protein G-conjugated carboxylate microsphere beads to stimulate proliferative responses of polyclonal short-term T cell lines from cattle immunised with purified *A. marginale* outer membranes [19]. Of note, both studies used T cell lines, rather than unpurified splenocytes or bulk peripheral blood mononuclear cells, and neither reported a systematic evaluation of the T cell screening process.

Herein, we present a series of *in vitro* and *in vivo* experiments designed to demonstrate proof-of-concept for high throughput identification of antigens recognised by T cell responses in human or murine systems, using IVTT products unpurified, affinity purified through nickel resin or magnetic beads, or absorbed in beads to enhance the cell mediated immunogenicity by promoting dendritic cell uptake [20], [21]. IVTT produced antigens of FluM and FluHA from Influenza A virus [22], CMVpp65 from Cytomegalovirus [23] and EBNA3A from Epstein-Barr virus [24] were assayed using bulk human PBMC for T cell recognition. Additionally, *Plasmodium yoelii* circumsporozoite protein (*Py*CSP) [25] IVTT products were assayed for antigenicity *in vitro* using splenocytes from *Py*CSP-immunized mice, and for immunogenicity *in vivo* as assessed by capacity to boost a *Py*CSP-specific immune response primed by plasmid DNA. In both human and murine systems, T cell responses were evaluated by IFN- γ ELISpot and Cytometric Bead Array (CBA) cytokine assays. Robust IFN-γ, TNF-α and IL-10 responses were detected, and IVTT products affinity purified through nickel resin or magnetic beads were highly effective. Considering the loss associated with purification, the nickel resin method proved the most optimal.

## Results

### Production and yield of IVTT products

**Figure 1:**
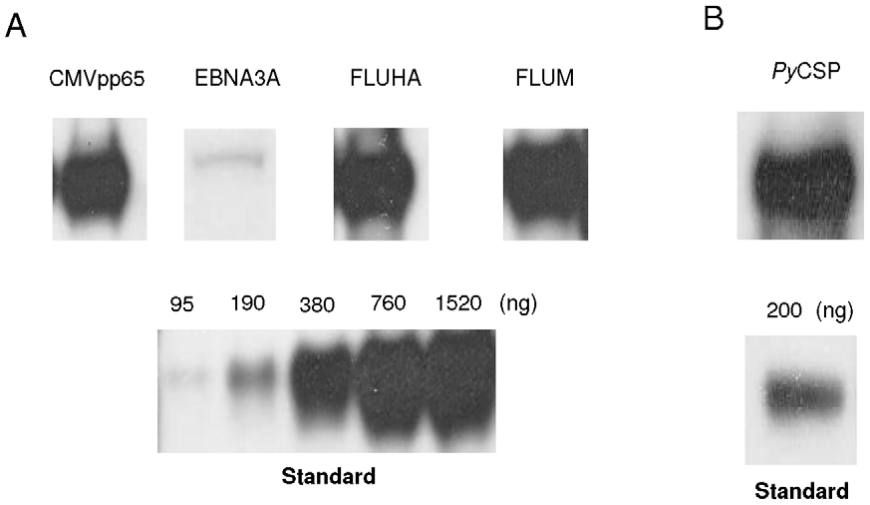
Recombinants produced using *E. coli* cell-free IVTT system. Western Blot and quantification of (A) viral antigens FluM, FluHA, CMVpp65 or EBNA3A and (B) parasite antigen PyCSP pIVEX HisHA IVTT products, probed with mAb against the C-terminal HA tag. Whole IVTT extracts (5 µl) of each antigen were run on a 12% NUPAGE gel, transferred to a PVDF membrane, and probed with anti-HA HRP antibody (1∶500 dilution). Protein expression was quantitated against an IVTT-produced recombinant *P. falciparum* (PF14_0051) protein of known concentration expressing the same N-terminal 6xHis and C-terminal HA tags. Yields for each full length antigen were as follow: FluM, 1.15 µg/ µl; FluHA, 0.73 µg/ µl; CMVpp65, 0.7 µg/ µl; EBNA3A, 4 ng/ µl; and PyCSP, 0.6 µg/ µl.

Coding sequences were expressed by coupled transcription-translation in the *E. coli* cell-free IVTT system. An aliquot of each reaction mixture was analysed by SDS-PAGE and western blot, followed by chemiluminescence of the membrane to visualize the products formed. All four viral (FluHA, FluM, CMVpp65, EBNA3) and one parasite (*Py*CSP) recombinants could be produced using the manufacturer's recommended conditions. The average yield of each full-length recombinant, detected using an anti-HA antibody directed against the C-Terminal HA tag, was as follow: FluM, 1.15 µg/µl; FluHA, 0.73 µg/µl (two moles of HA tag per molecule of FLU-HA); CMVpp65, 0.7 µg/µl; EBNA3A, 4 ng/µl; and *Py*CSP, 0.6 µg/µl (Figures 1A and B and Figure S1A). Western blot analysis using an antibody directed against the N-Terminal 6xHIS tag identified the presence of partial products for each viral recombinant (*Py*CSP not tested) (Figure S1B). Solubility analysis showed that a high proportion (at least 70%) of each recombinant was insoluble. A series of optimization studies including kinetics of expression (3 hr to 6 hr), reaction temperature (16°C, 25°C, 30°C), speed (stationary, 300 rpm, 600 rpm), addition of protease inhibitor cocktails, or addition of non-ionic detergents to promote protein solubilisation (Triton X-110 or Triton X-114) had no significant effect on the yield of full-length protein or partial products, or protein solubility (data not presented). Accordingly, a standard protocol of 4 hrs incubation at 30°C and 300 rpm was adopted for the IVTT reactions. IVTT products were used either unpurified (whole extract), associated to Polybeads or ProteinG beads, or purified using NI-NTA resin or MagneHis Ni-particles. The MagneHis purification method was associated with a very low recovery yield and this yield was much lower than with the NI-NTA system. For example, for *Py*CSP, the loss associated with purification using Ni-NTA or MagneHis was approximately 30% and 80% respectively.

**Figure 2:**
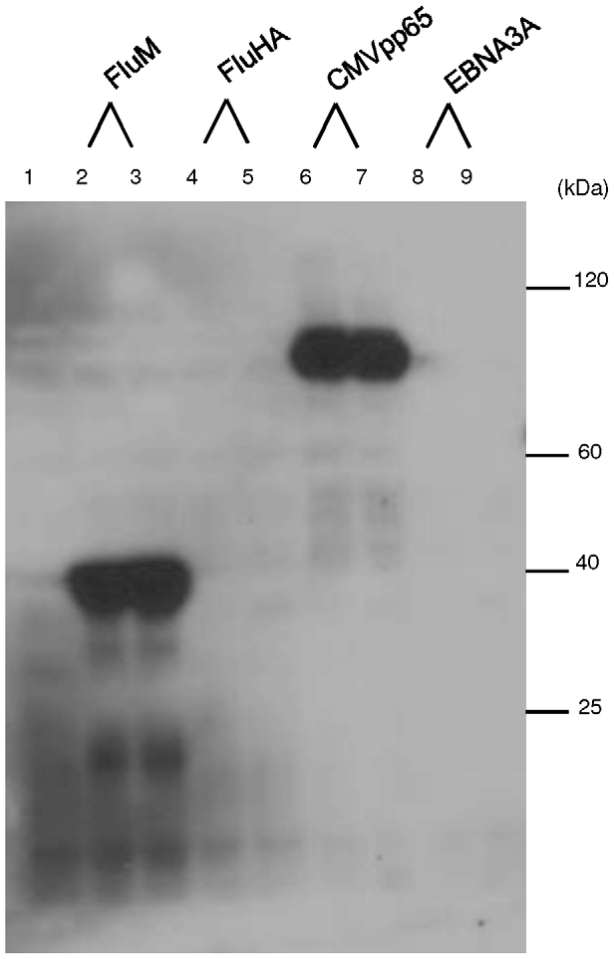
Recombinants produced using the insect cell extract IVTT system. Western Blot of viral antigen pIVEX HisHA IVTT products probed with anti-HA HRP antibody (1∶500). Lane 1: negative control IVTT control reaction. Lanes 2 and 3: FluM, supernatant and whole extract. Lanes 4 and 5: FluHA supernatant and whole extract. Lanes 6 and 7: CMVpp65 supernatant and whole extract. Lanes 8 and 9: EBNA3A supernatant and whole extract. FluM and CMVpp65 were expressed efficiently and as soluble proteins using this system. FluHA and EBNA3A were not expressed. *Py*CSP, not tested.

We also evaluated production of the viral antigens in an insect cell based cell-free system (*Py*CSP not tested). In a limited number of attempts using the manufacturer's recommended conditions, only two of the four viral protein targets (FluM and CMVpp65) could be produced in this eukaryotic system. However, in contrast to results with the *E. coli* system, solubility analysis showed that at least 90% of the recombinants were soluble and partial polypeptides were not detected (Figure 2).

In summary, all FluHA, FluM, CMVpp65, EBNA3 and *Py*CSP IVTT recombinants could be produced in the *E. coli* cell-free IVTT system, using the manufacturer's recommended conditions. However, at least 70% of each IVTT product was insoluble. In contrast, only two of the four viral antigens could be produced in the insect cell based system but in this system at least 90% of the product was soluble.

### *In vitro* stimulation of PyCSP antigen-specific T cell response using IVTT products

The ability of IVTT products to stimulate an antigen-specific T cell response *in vitro* was evaluated in the *P. yoelii* CSP model. Splenocytes from mice (n = 5/group) immunized with VR2516 *Py*CSP plasmid DNA or VR1020 control plasmid were stimulated *in vitro* with unpurified r*Py*CSP IVTT; r*Py*CSP IVTT associated to Polybeads or ProteinG beads; r*Py*CSP IVTT purified using NI-NTA resin, MagneHis Ni-particles, or anti-HIS; or synthetic peptides representing defined T cell epitopes from *Py*CSP. Antigen-specific cytokine production was assayed using IFN-γ ELISpot or CBA assays.

**Figure 3:**
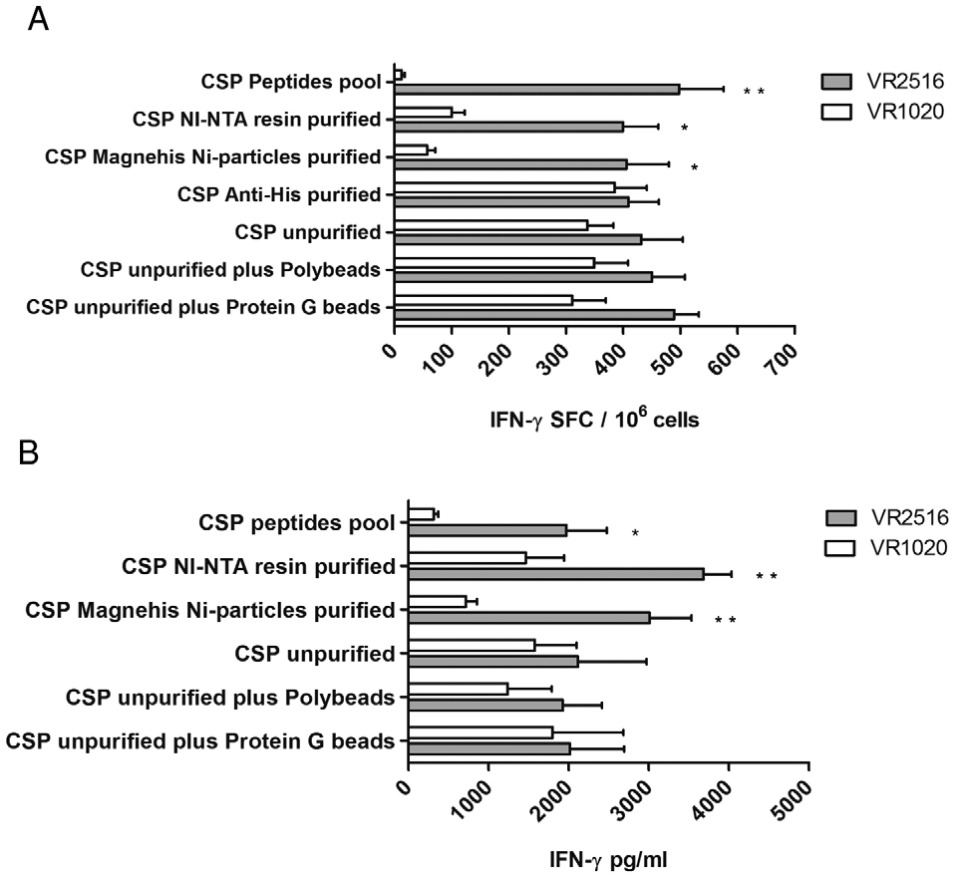
*In vitro* evaluation of IVTT proteins. *Py*CSP specific IFN-γ secretion by splenocytes of mice immunized with VR2516 *Py*CSP plasmid DNA or VR1020 control DNA. Splenocytes were cultured with unpurified r*Py*CSP IVTT; r*Py*CSP IVTT associated to Polybeads or ProteinG beads; r*Py*CSP IVTT purified using NI-NTA resin, MagneHis Ni-particles, or anti-HIS; or synthetic peptides representing defined T cell epitopes from PyCSP (positive control), as indicated. (A) IFN-γ ELISpot responses (spot forming cells, SFC per million splenocytes) of cultured splenocytes, analysed after 36 hrs stimulation. (B) secreted IFN-γ in culture supernatant, measured by Cytometric Bead Array (CBA) after 48 hrs stimulation. Data are presented as mean +/- standard deviation of 5 mice/group. *P<0.05 and **P<0.01 compared to VR1020 control.

The number of IFN-γ SFCs was significantly higher with splenocytes from VR2516 immunized mice as compared to VR1020 immunized mice when stimulated *in vitro* with *Py*CSP IVTT purified using NI-NTA resin (*p* = 0.013), MagneHis Ni-particles (*p* = 0.015) or PyCSP synthetic peptides (*p* = 0.003) (Figure 3A). There was no significant difference when splenocytes were stimulated with either unpurified IVTT or unpurified IVTT associated to Polybeads or Protein G beads (Figure 3A). Unexpectedly, the use of wells pre-coated with anti-HIS mAb to capture the tagged proteins was poorly effective with no difference noted between the VR2516 and VR1020 groups. Markedly higher background reactivity was noted with the unpurified or bound IVTT preparations as compared to the purified preparations, presumably due to the presence of high levels of LPS and proteins in the *E. coli* extract (Figure 3A).

Consistent with the ELISpot data, the amount of secreted IFN-γ by CBA in cultures of splenocytes from VR2516 versus VR1020 immunized mice was significantly greater in cultures stimulated *in vitro* with *Py*CSP IVTT purified using NI-NTA resin (*p* = 0.005) or MagneHis Ni-particles (*p* = 0.003) or *Py*CSP synthetic peptides (*p* = 0.02) (Figure 3B). There was no significant difference when splenocytes were stimulated with either unpurified IVTT or unpurified IVTT associated to Polybeads or Protein G beads. No significant antigen-specific IL-10, TNF-α or IL-6 responses could be detected upon stimulation with purified or unpurified IVTT products; responses were detected upon stimulation with *Py*CSP peptides (Figures S2A and S2B; data not presented for IL-6).

In summary, robust antigen-specific IFN-γ responses from splenocytes of mice immunized with *Py*CSP plasmid DNA could be induced following *in vitro* stimulation with IVTT products purified by either NI-NTA resin or MagneHis Ni-particles, but not by unpurified IVTT reactions or IVTT products associated to Polybeads or Protein beads. There was no significant difference between responses induced by NI-NTA resin and MagneHis Ni-particle purifications, but there was a much greater loss of recombinant associated with purification using the MagneHis Ni-particles as compared to NI-NTA resin. In general, responses induced by stimulation with the purified *Py*CSP IVTT products were comparable to responses induced by stimulation with a pool of synthetic peptides representing defined T cell epitopes from *Py*CSP.

### *In vivo* stimulation of PyCSP antigen-specific T cell response using IVTT products

We next evaluated the capacity of unpurified and purified IVTT products to boost *Py*CSP primed immune responses *in vivo*. Mice (n = 5/group) were primed with VR2516 *Py*CSP plasmid DNA and then boosted in vivo with unpurified r*Py*CSP IVTT, *Py*CSP IVTT associated to Polybeads or ProteinG beads, or *Py*CSP IVTT purified using NI-NTA resin or MagneHis Ni-particles, all adjuvanted with Alum. Splenocytes were stimulated *in vitro* with A20 target cells transfected with VR2516 *Py*CSP DNA or A20 cells pulsed with *Py*CSP synthetic peptides. Antigen-specific cytokine production was assayed using IFN-γ ELISpot or CBA assays.

**Figure 4:**
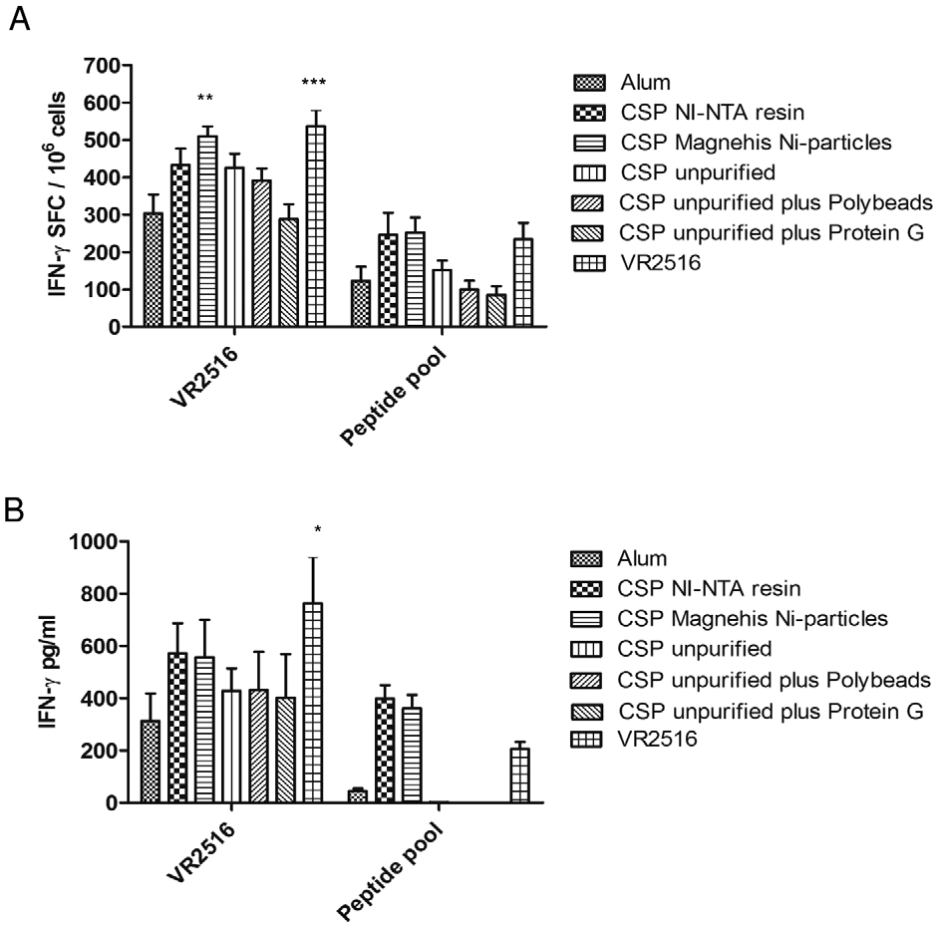
*In vivo* evaluation of IVTT proteins. *Py*CSP specific IFN-γ responses of mice immunized with VR2516 *Py*CSP plasmid DNA and boosted *in vivo* with IVTT products: unpurified r*Py*CSP IVTT; r*Py*CSP IVTT associated to Polybeads or ProteinG beads; or r*Py*CSP IVTT purified using NI-NTA resin or MagneHis Ni-particles. All IVTT products were formulated with Alum adjuvant. Parallel groups of mice were boosted with either Alum only or VR2516 as controls. Splenocytes were cultured with A20 cells transfected with VR2516 *Py*CSP plasmid DNA or A20 cells pulsed with synthetic peptides representing defined *Py*CSP T cell epitopes. A) IFN-γ ELISpot responses (spot forming cells, SFC per million splenocytes) of cultured splenocytes, analysed after 36 hrs stimulation. (B) secreted IFN-γ in culture supernatant, measured by Cytometric Bead Array (CBA) after 48 hrs stimulation. Data are presented as mean +/- standard deviation of 5 mice/group. *P<0.05, **P<0.01 and ***P<0.001 compared to negative control (Alum only boost).

IFN-γ responses were detected by both ELISpot and CBA following *in vivo* boosting with *Py*CSP DNA (*p*<0.001 for ELISpot and *p*<0.05 for CBA) or *Py*CSP IVTT purified using either NI-NTA resin or MagneHis Ni-particles (*p*<0.01 for ELISpot), as compared to Alum control (Figures 4A and 4B). Responses stimulated by purified IVTT products were almost as robust as those stimulated with *Py*CSP DNA. Low responses were noted when mice were boosted with unpurified whole extract or unpurified IVTT associated to either Polybeads or ProteinG beads.

Significant IL-10 responses were also detected following boosting with *Py*CSP IVTT purified using either NI-NTA resin (*p*<0.001) or MagneHis Ni-particles (*p*<0.001), as compared to Alum control, but not following boosting with unpurified whole extract or IVTT products associated to either Polybeads or ProteinG beads, or with *Py*CSP DNA (Figure S2C). TNF-α responses were not significantly different from the controls for any of the conditions (Figure S2D). IL-2 responses were induced only by purified IVTT products (data not presented). No significant responses were detected for other cytokines assayed using the CBA assay.

In summary, consistent with the results following *in vitro* stimulation with IVTT products, robust antigen-specific IFN-γ responses could be induced by *in vivo* boosting with *Py*CSP IVTT products purified by either NI-NTA resin or MagneHis Ni-particles, but not by unpurified IVTT reactions or IVTT products associated to Polybeads or ProteinG beads. There was no significant difference between purification with either NI-NTA resin or MagneHis Ni-particle, nor between purified IVTT products and plasmid DNA. The magnitude of Th1 (IFN-γ and TNF-α) responses stimulated *in vitro* by purified IVTT *Py*CSP products were similar to those stimulated by *Py*CSP plasmid DNA.

### *In vivo* stimulation of PyCSP antigen-specific antibody response using IVTT products

**Figure 5:**
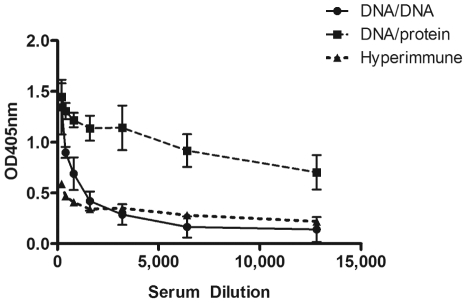
Antibody response induced by *Py*CSP IVTT protein. *Py*CSP specific antibody responses of mice immunized with VR2516 *Py*CSP plasmid DNA and boosted *in vivo* with VR2516 or r*Py*CSP IVTT purified using NI-NTA resin and formulated with Alum adjuvant. Serially diluted sera samples were assayed against recombinant PyCSP capture antigen. Data are presented as OD450 at each dilution, mean +/- standard deviation of 5 mice/group.

The ability of IVTT products to stimulate an antigen-specific antibody response was also determined. Sera from mice (n = 5/group) primed with VR2516 *Py*CSP plasmid DNA and boosted *in vivo* with r*Py*CSP IVTT purified using NI-NTA resin plus Alum were evaluated by ELISA against synthetic peptide representing the recombinant *Py*CSP protein. Data demonstrated that *in vivo* boosting with PyCSP IVTT products significant boosted antigen-specific antibody responses relative to responses induced by PyCSP plasmid DNA in the absence of boosting (Figure 5).

### *In vitro* stimulation of viral antigen-specific T cell responses by human PBMCs using IVTT products

The antigenicity of IVTT products in human PBMC cultures was next evaluated. The viral antigens FluM, FluHA, CMVpp65 and EBNA3A produced by IVTT were used unpurified, associated to Polybeads or ProteinG beads, or purified using NI-NTA resin or MagneHis Ni-particles to assay recall cytokine responses from PBMCs of 10 healthy humans by IFN-γ ELISpot or CBA.

**Figure 6:**
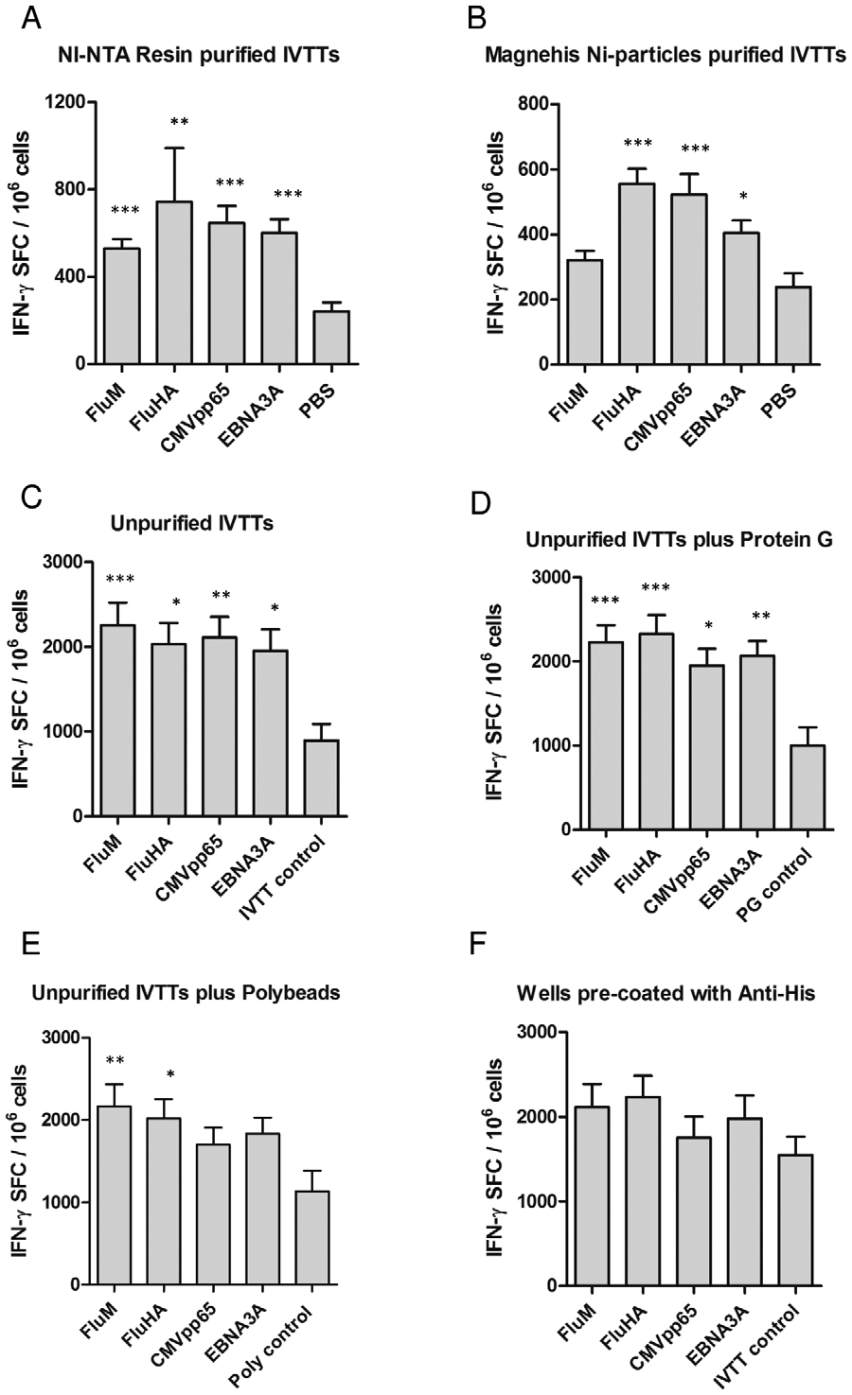
Antigen-specific IFN-γ ELIspot responses by human PBMC stimulated with IVTT-proteins. PBMCs were cultured with IVTT-produced FluM, FluHA, pp65 and EBNA3A purified using (A) NI-NTA nickel resin or (B) MagneHis Ni-particles; (C) unpurified; associated to (D) ProteinG beads or (E) Polybeads; or (F) added to wells precoated with Anti-His. Negative controls were medium only, unpurified whole extract, and whole extract associated to Polybeads or Protein G beads; positive control was CEF peptide pool. IFN-γ ELIspot responses (spot forming cells, SFC) of cultured PBMCs were analysed after 36 hrs stimulation. Data are presented as mean +/- standard deviation of 10 volunteers. *P<0.05, **P<0.01 and ***P<0.001 compared to negative controls.

Consistent with the results in murine system, significant IFN-γ ELISpot responses (p<0.05) were detected with all IVTT antigens purified using NI-NTA resin (Figure 6A) and for all antigens purified using MagneHis Ni-particles except FluM (Figure 6B) (as compared to PBS); the lack of responses with FluM was attributed to a low yield post MagneHis Ni purification (data not presented). Unexpectedly, significant IFN-γ ELISpot responses were also noted with all IVTT viral antigens used unpurified (compared to empty pIVEX HisHA IVTT) (Figure 6C) or associated to ProteinG beads (compared to empty pIVEX HisHA IVTT associated with ProteinG beads) (Figure 6D), but only for only two or the four IVTT viral antigens associated with Polybeads (FluM and FluHA; compared to empty pIVEX HisHA IVTT associated with Polybeads) (Figure 6E).

Also consistent with the murine system, no significant responses were detected for any antigens when the ELISpot wells were pre-coated with anti-His mAb to capture the His-tagged IVTT proteins. The background responses with the unpurified or bound IVTT products were greater than for purified IVTT products, presumably as a result of prior exposure of the human subjects to *E. coli* bacteria or presence of LPS. Significant IFN-γ responses to the CEF peptide pool *p*<0.0001, compared to PBS) were noted for all subjects (data not presented).

**Figure 7:**
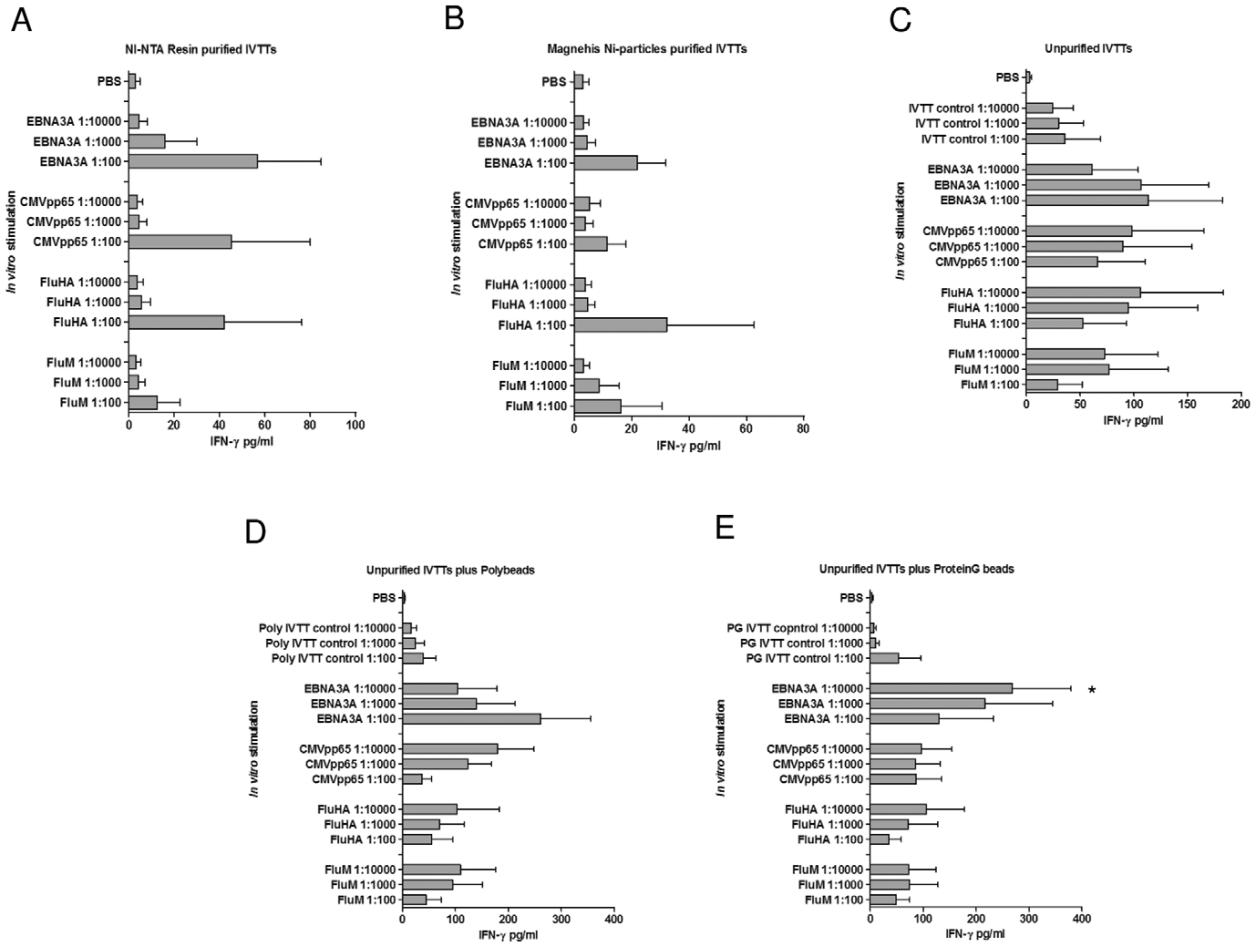
Antigen-specific IFN-γ CBA responses by human PBMC stimulated with IVTT-proteins. PBMCs were cultured with IVTT-produced FluM, FluHA, CMVpp65 and EBNA3A purified using (A) NI-NTA nickel resin or (B) MagneHis Ni-particles; (C) unpurified; associated to (D) Polybeads or (E) ProteinG beads; diluted 1∶100, 1∶1000 or 1∶10,000. Negative controls were medium only, unpurified empty pIVEXHisHA IVTT extract, and empty pIVEXHisHA IVTT extract associated to polybeads or protein G beads. Positive controls were CEF peptide pool or PHA (data not shown). Secreted IFN-γ in the supernatant of cultured PBMCs was analysed by Cytometric Bead Array after 72 hrs stimulation. * *P*<0.05 compared to negative controls.

For all viral antigens, robust IFN-γ responses were detected by CBA in cultures stimulated at 1∶100 dilution with IVTT products purified using NI-NTA resin (Figure 7A) or MagneHis Ni-particles (Figure 7B) but not with purified products at 1∶1,000 or 1∶10,000 dilutions, presumably due to the low amount of antigen in culture (Figures 7A and 7B). With unpurified IVTT products or IVTT antigens associated to Polybeads or ProteinG beads, IFN-γ responses were generally better at the higher dilution (1∶10,000>1∶1,000>1∶100) where background *E. coli* responses were lower (Figures 7C–E). However, none of these IFN-γ responses were statistically different from the controls due to variations in the cytokine levels between individuals (*p*<0.05 only for EBNA3A-Protein G beads at 1∶1000).

The profile of TNF-α responses by CBA was very similar to that of IFN-γ (Figure S3). For all viral antigens (except FluM-Ni-NTA), TNF-α responses were statistically significant compared to controls for cultures stimulated at 1∶100 dilution with IVTT products purified using NI-NTA resin (Figure S3A) or MagneHis Ni-particles (Figure S3B). Responses were also significant at 1∶1,000 dilutions for CMVpp65 and EBNA3 all purified using NI-NTA resin or MagneHis Ni-particles; and FluHA purified with MagneHis Ni-particles. The TNF-α profile was also similar to that of IFN-γ for unpurified and bead-associated IVTT products, with the highest responses almost always detected at 1∶10,000 (except for EBNA3) (Figures S3C-E). However, none of these TNF-α responses were statistically significant (except for unpurified CMVpp65 at 1∶10,000), as noted above for IFN-γ. The overall level of TNF-α was enhanced by association to Polybeads and Protein G beads, relative to unpurified IVTT products (up to 7-fold with EBNA3A-Polybeads at 1∶100 dilution).

The profile of IL-10 responses by CBA was very similar to that of IFN-γ and TNF-α (Figure S4) for cultures stimulated with IVTT products purified using NI-NTA resin (Figure S4A) or MagneHis Ni-particles (Figure S4B) with the best responses detected at 1∶100 (Figure S4). However, for unpurified or bead-associated IVTT products, the IL-10 profile was inverse to that of IFN-γ and TNF-α with the best responses at a 1∶100 dilution (Figures S4C-E), consistent with potential immunosuppressive effects of IL-10. Significant response was found only in EBNA3A 1∶100 purified with MagneHis Ni-particles. As noted for TNF-α, the overall level of IL-10 was enhanced by association to Polybeads and Protein G beads relative to unpurified IVTT products (up to 7-fold with EBNA3A-Polybeads at 1∶100 dilution). Responses for other cytokines assayed by CBA were not significant (data not presented).

In summary, consistent with the murine data, robust antigen-specific IFN-γ and TNF-α responses from human PBMCs could be induced following *in vitro* stimulation with IVTT products purified by either NI-NTA resin or MagneHis Ni-particles. In contrast to the murine system, positive IFN-γ and TNF-α responses to some antigens could also be induced by unpurified IVTT reactions or IVTT products associated to Polybeads or Protein beads, indicating that the *E. coli* and LPS background of the whole extract was not sufficient to mask the immunogenicity generated by the IVTT recombinant products. For some but not all of the evaluated antigens, association of IVTT products to Polybeads or Protein G beads enhanced the antigenicity about 2-fold to 4-fold.

## Discussion

The rapidly growing amount of genetic and proteomic information available in the post-genomic era precludes the use of traditional cell-based protein expression systems to screen proteins of interest for their potential as vaccine targets. The development of cell-free based systems, first described in 1960's [26], allows the high throughput recombinant expression of thousands of proteins in reduced volume and time. The most popular cell-free systems are based in *E. coli*, wheat germ and rabbit reticulocytes extracts [4], [5], [27]. This platform has been applied in a number of proteomics-based studies including structural and functional proteomics [28], [29], [30], protein evolution [31], unnatural amino acids and protein labeling [32], [33], protein interaction [34], diagnostics and therapeutics [35], [36] and protein microarrays [15], [37], [38], [39]. The protein microarray platform, exploiting antigen-specific antibodies present in plasma or sera from exposed or immunized animals or humans, has been of particular interest to our laboratory to identify potential target antigens for malaria vaccine development [10], [11], [40]. Although *Plasmodium* proteins have proven particularly difficult to express using conventional cell-based methods, efficient expression of *P. falciparum* proteins (≥ 93%) has been obtained using the *E. coli* cell-free system [11]. The wheat germ system has been also used for production of *P. falciparum* proteins, but with less efficiency (75%) [12].

The success with cell-free protein expression suggest that this system could be also applied to cellular screening, to identify antigenic targets of T cell responses from genomic sequence data. However, this has not yet been adequately explored despite that T cells play a central role in orchestrating acquired immunity against infectious diseases [41], [42]. Both CD8^+^ and CD4^+^ T cells can mediate their effector function directly via cytotoxicity or indirectly via cytokines. CD4^+^ T cells can also provide help for CD8^+^ T cells or can recruit and activate B cells for antibody secretion. To date, the only reports have been restricted to measurement of proliferative T cell responses in PBMCs of smallpox vaccines or of cattle immunized with a purified *Anaplasma marginale* outer membrane preparation to a small number of IVTT produced vaccinia virus proteins or outer membrane proteins, respectively [18], [19], [43]. Neither of those studies comprehensively assessed and optimized the application of IVTT products for large-scale or proteome-wide cellular screening. Accordingly, herein, we report proof of concept for the potential of this approach a strategy for proteome-wide identification of antigens targeted by cell mediated immunity in both viral and parasite models, using IVTT products with specimens from human and mice.

Our human studies used well characterized antigens from EBV, CMV and Influenza A virus. which are known to be targeted by both CD4^+^ and CD8^+^ T cell responses and with defined T cell epitopes [44]. The murine studies used the well characterized sporozoite coat protein, the circumsporozoite protein (CSP), from the *P. yoelii* rodent malaria parasite. We tested each IVTT antigen individually and presented to T cells in different forms – either unpurified, purified using NI-NTA resin or MagneHis Ni-particles, or associated to Polybeads or ProteinG beads. Synthetic peptides representing defined T cell epitopes from the respective antigens were assayed in parallel as positive controls. The primary immune readout was IFN-γ production due to its crucial role in protection or pathogenesis of complex diseases [45].

For all four viral and one parasite antigens tested in this study, partial products were common. The production of partial fragments in *E. coli* based IVTT reactions has been described previously and attributed to a phenomena called translational pausing but the presence of partial products did not adversely affect immunogenicity or antigenicity [46]. Since the antigens produced by *E. coli* based IVTT were at least partially insoluble, simple techniques for purification, such as antibody coated beads, were not effective. We therefore explored alternative purification options including Ni-NTA affinity resin and MagneHis Ni-particles. Both purification systems tested using denaturating conditions proved efficient but a much higher product recovery was obtained with the Ni-NTA resin as compared to the MagneHis Ni-particles. Unexpectedly, with the eukaryotic based insect cell-free system, the solubility of the recombinants increased to almost 100%, but only two of the four proteins expressed in the *E. coli* cell-free system could be expressed in the insect cell based system, at least for the limited number of times expression was attempted. These data suggest that further studies with the insect cell-free system are warranted. Newer technologies of high throughput purification such as affinity ZipTips (Millipore, Ireland) and Ni-NTA plates (Qiagen, Valencia, CA) can facilitate the purification process, but do not effectively deal with solubility issues.

In both human/virus and murine/parasite models, IVTT products purified by either NI-NTA resin or MagneHis Ni-particles were highly effective inducers of antigen-specific IFN-γ and TNF-α responses *in vitro*. In the human/virus but not murine/parasite model, antigen-specific IFN-γ and TNF-α responses could also be induced by unpurified IVTT reactions or IVTT products associated to Polybeads or Protein beads, indicating that the *E. coli* and LPS background of the whole extract was not sufficient to mask the immunogenicity of the IVTT recombinant products. Addition of carboxylate beads to unpurified IVTT products increased the immunogenicity of the IVTT viral products by up 1.8-fold for IFN-γ, 2.6-fold for IL-10 and 7-fold for TNF-α. The most robust and specific antigen-specific IFN-γ and TNF-α responses (by CBA) were detected at the highest dilution of IVTT product, where background *E. coli* responses were lowest. This is a favorable scenario for proteomic-wide scale cellular screening, as the use of highly diluted IVTT products is more cost-effective. Unexpectedly, poor results were obtained with ELIspot wells pre-coated with anti-IFN-γ mAb as well as anti-HIS mAb to bind the HIS tag on the IVTT product. *In vivo* studies with *Py*CSP IVTT products confirmed that the target protein was produced and that the IVTT produced proteins were immunogenic.

These data demonstrate the potential of IVTT products as a useful tool for the proteome-wide screening of cellular targets of viral, parasitic or bacterial immunity Overall, IVTT products affinity purified through nickel resin or magnetic beads proved the most efficient inducers of sensitive and specific antigen-specific cytokine responses, the nickel resin method was associated with the greater yield post-purification. Although not specifically evaluated herein, it is likely that such cell-free approaches may be suited to the identification of targets of CD4^+^ T cell responses, but not targets of CD8^+^ T cell responses due to a requirement for target antigen processing and presentation [43], [47], [48]. Rather, epitope-based approaches based on prediction of high affinity binding class I T cell epitopes using computerized algorithms, such as that reported by us previously [49] are probably more appropriate.

Overall, the work reported here provides proof of concept for the potential for high-throughput identification from genomic sequence data of antigenic targets of T cell responses from complex pathogens which threaten public health. Such antigens may represent promising candidates for the development of vaccines that have thus far proved elusive.

## Materials and Methods

### Human subjects

Ten healthy adult Caucasian volunteers (five male and five female; mean age 36.3±7.1 years old) were recruited with written informed consent under a protocol (P1111) approved by the Queensland Institute of Medical Research Human Research Ethics Committee. All studies with human specimens were approved by the Queensland Institute of Medical Research Human Research Ethics Committee (P1111) and conducted in compliance with all applicable regulations governing protection of human subjects. Although the history of vaccination in these subjects has not been documented, it is likely that all would have been vaccinated against or exposed to influenza, EBV, and CMV, given the documented prevalence of these viruses in human populations; and all were known to respond to the CEF peptide pool which comprises CD8^+^ T cell epitopes from FLU, EBV and CMV.

### Animals

Female BALB/c (H-2^d^) mice aged 6-8 weeks were purchased from The Animal Resource Centre (Perth, WA) and maintained under standard conditions. All studies were approved by the Queensland Institute of Medical Research Animal Ethics Committee (protocol P1111).

### Plasmid DNA

The plasmid DNA vaccines encoding the *P. yoelii* circumsporozoite protein (CSP; VR2516) and the control plasmid (VR1020) have been previously described [50], [51]. The DNA vaccines were prepared using the EndoFree Plasmid Mega Kit (Qiagen, Valencia, CA) according to manufacturer's instructions and administered in sterile saline.

### Protein expression vector

A custom vector was developed for protein expression in the *E. coli* cell-free transcription translation system. This vector, called pIVEX HisHA AmpR, was modified from the commercially available pIVEX 2.4d and pIVEX 2.5d vectors (Roche Applied Science, Mannheim, Germany) by incorporating both the N-terminal HIS tag and C-terminal HA tag into the one vector backbone. The HA tag was released from pIVEX 2.5d by digestion with *Bam*HI and *Xma*I restriction enzymes (New England Biolabs, Ipswich, MA) for 4 hrs at 37°C (1 µg DNA/1 unit enzyme, 20 µl volume) and the fragment then extracted from a 3% agarose gel and purified using a commercially available QIAquick PCR purification kit (Qiagen, Germany) according to manufacturer's instructions. The purified HA-tag was ligated overnight at 16°C into *Bam*HI and *Xma*I digested pIVEX 2.4d vector using 400 units T4 ligase (New England Biolabs), 25 ng of linearized pIVEX 2.4d vector and 70 ng of the HA-tag insert in a 20 µl reaction. The ligated product was purified from a 1% agarose gel as described above, transformed into TOP10 thermocompetent cells (Invitrogen), and grown on LB plates in the presence of 100 µg/ml ampicillin. Positive colonies were screened by colony PCR using 0.2 units/µl Expand Taq polymerase in Buffer 2 (Roche Diagnostics), 0.4 µM dNTPs and 0.4 µM each primer (forward: TAATACGACTCACTATAGGG; reverse TGCTAGTTATTGCTCAGCGG) using the following conditions: initial denaturation at 95°C for 5 min; 35 cycles at 95°C for 30 sec, 55°C for 30 sec and 68°C for 1 min; and a final extension at 68°C for 10 min.

### Recombinant protein constructs of FluM, FluHA, CMVpp65, EBNA3A and PyCSP

For cloning the target genes into the customized pIVEX HisHA vector, the complete open read frames of the Influenza matrix protein (FluM; Influenza A virus A/Puerto Rico/8/34 strain, accession #AF389121), Influenza haemagglutinin protein (FluHA; Influenza A virus A/Puerto Rico/8/34 strain, accession #AF389121), Cytomegalovirus phosphoprotein 65 (CMVpp65; human herpesvirus 5 strain AD169, accession #P06725), Epstein Barr Virus nuclear antigen 3A (EBNA3A; human herpesvirus 4 type 1 B95-8 accession #NC_007605) and *P. yoelii* circumsporozoite protein (*Py*CSP; strain 17XNL, accession #J02695) were amplified by PCR using gene-specific primers flanked with restriction enzyme sites (Table 1). The 50 µl PCR reaction contained 0.2 units/µl Expand Taq polymerase in Buffer 2 (Roche diagnostics), 0.4 µM dNTPs and 0.4 µM each primer and 50 ng DNA template. PCR conditions were: initial denaturation at 95°C for 5 min; 45 cycles at 95°C for 30 sec, 55°C for 30 sec and 68°C for 3 min; and a final extension at 68°C for 10 min. The fragments corresponding to the expected size were excised from a 1% agarose gel, purified using the QIAquick PCR purification kit (Qiagen, Germany), and digested overnight at 37°C using the restrictions enzymes for which the cutting site was present in the flanking sequence. The pIVEX HisHA vector was also digested overnight at 37°C with the same set of restriction enzymes. After cleanup using the PCR purification kit, the gene fragments and the linearized vector were ligated overnight at 16°C at an insert∶vector ratio of 1∶20 using 30 ng of vector and 400 units T4 ligase (New England Biolabs) in a 20 µl reaction volume. The ligation product was transformed into TOP10 thermo-competent cells, grown overnight at 37°C on LB ampicillin plates (100 µg/ml), and colonies screened by PCR using the same protocol for the customized vector, except that the extension time of the colony PCR was increased to 3 min. One positive colony of each construct was grown overnight at 37°C in LB containing ampicillin (100 µg/ml) and the plasmid purified using the QIAprep Spin Miniprep kit (Qiagen) according to manufacturer's instructions. In a few instances where the IVTT production was poor, the DNA was further purified using phenol/chloroform extraction and ethanol precipitation which improved the yield, but this was not routinely done. All constructs were confirmed by DNA sequencing before use in the IVTT reactions.

**Table 1:** DNA sequences and primers.

Antigen	GenBank accession number	Strain/Source	Ref	Restriction enzyme /Forward primer	Restriction enzyme /Reverse primer
FluM	AF389121.1	Puerto Rico/ H1N1 segment 7	[22]	*Not*I / 5′ GGGGCGGCCGCATGAGTCTTCTAACCGAGG 3′	*Sal*I / 5′ GGGGTCGACGCTTGAACCGTTGCATCTG 3′
FluHA	AF389118.1	Puerto Rico/H1N1 segment 4	[22]	*Not*I / 5′ GGGGCGGCCGCATGAAGGCAAACCTACTGG 3′	*Sal*I / 5′ CAGTGCAGAATATGCATCCGTCGACCCC 3′
pp65	FJ527563.1	AD169/Human herpesvirus 5	[23]	*Not*I / 5′ GAAGCGGCCGCATGGAGTCGCGCGGTCGCCGTTG 3′	*Xho*I / 5′ GGGCTCGAGACCTCGGTGCTTTTTGGGC 3′
EBNA3A	V01555.2	B95-8/Epstein-Barr virus	[24]	*Not*I / 5′AAGGAAAAAAGCGGCCGCATGGACAAGGACAGGCCG 3′	*Sal*I / 5′ GGGGTCGACGGGCCTCATCTGGAGGAT 3′
*Py*CSP	FJ527563.1	L/*Plasmodium yoelii*	[25]	*Not*I / 5′GGCCGCGGCCGCCTTCCAGGATATGGACAA 3′	*Sal*I / 5′ TCGAGTCGACTATTAAAGAATACTAATAC 3′

Footnote to Table 1: The complete open read frames of genes encoding the Influenza A virus matrix (FluM) or haemagglutinin (FluHA), Cytomegalovirus phosphoprotein 65 (CMVpp65), Epstein Barr Virus nuclear antigen 3A (EBNA3A) and the *Plasmodium yoelii* circumsporozoite surface protein (*Py*CSP) were cloned into pIVEX His HA vector using gene-specific forward and reverse primers containing specific restriction enzyme sites, as indicated. The source of each template and the corresponding GenBank accession numbers are also listed.

### Recombinant protein expression

Recombinant proteins were synthesized by cell-free *in vitro* transcription and translation using the Rapid Translation System 100 *E. coli* HY kit (Roche Diagnostics) or EasyXpress Insect Cell Protein kit (Qiagen) according to the manufacturer's instructions. In some experiments, protease inhibitor cocktails (Cat P2714, Sigma-Aldrich, St Louis, MO; Cat 1836170, Roche Diagnostics), or addition of non-ionic detergents (Triton X-110 or Triton X-114) were added according to manufacturer's recommended protocol: 4 hr at 25°C. The recombinant antigens were stored at −20°C and used as immunogens for *in vitro* and *in vivo* up to one week after production. Protein expression of the full-length product (as evidenced by immunoblot using the anti-HA C-terminal tag mAb) was quantitated against an IVTT-produced recombinant *P. falciparum* (PF14_0051) protein of known concentration expressing the same N-terminal 6xHis and C-terminal HA tags. Immunoblot images were acquired in TIF format and analysed using the AnalySIS LS software (version 5.0; Soft Imaging Systems GmbH, Germany).

### Purification of IVTT recombinant products

The *E. coli* IVTT products were purified using two different methods based on affinity to the N-terminal 6xHIS tag. One method used mini-spin columns with a cellulose acetate filter (Pierce, Rockford, IL) and NI-NTA resin (Qiagen). For that, 200 µl of NI-NTA resin was added to a spin column connected to a collection tube. The column was washed twice with 600 µl of milliQ water and twice with 600 µl of Binding Buffer (50 mM NaH_2_PO_4_, 300 mM NaCl, 20 mM Imidazole and 8 M Urea pH 8) using centrifugation at 1500 rpm for 2 min. Then 50 µl of IVTT reaction was mixed with 700 µl of Binding Buffer and incubated at 4°C for 20 min in an orbital mixer, to solubilize the inclusion bodies. The solubilized sample was added to a NI-NTA resin column and incubated at 4°C for 30 min in an orbital mixer to maximize protein binding to the resin. The flow-through was removed via centrifugation at 1500 rpm for 2 mins and the column washed three times with 700 µl of Binding Buffer. The recombinant was eluted with 200 µl of Elution Buffer (50 mM NaH_2_PO_4_, 300 mM NaCl, 250 mM Imidazole and 8 M Urea pH 8) incubated at 4°C for 20 min in an orbital mixer before collection via centrifugation.

The second method of purification used the commercially available MagneHis Protein Purification System (Promega, Madison, WI) according to the manufacturer's instructions. For that, 50 µl of IVTT reaction was mixed with 700 µl of Binding Buffer (100 mM HEPES, 20 mM Imidazole and 8 M Urea pH 7.5) and incubated at 4°C for 20 min in an orbital mixer, to solubilize the inclusion bodies. Then, 30 µl of MagneHis Ni-particles was added to the sample and the mixture incubated at room temperature for 2 min. The sample tubes were connected to the MagneSphere Magnetic Separation Stand (Promega) and, after 30 sec of binding, the flow through was removed and discarded. The magnetic beads with bound protein were washed 3 times with 700 µl of Binding Buffer and the recombinant eluted in 200 µl of Elution Buffer (100 mM HEPES, 500 mM Imidazole and 8 M Urea pH 7.5) incubated at 4°C for 20 min in an orbital mixer before collection.

The eluted recombinants from both methods were dialyzed against PBS (desalting step) using Amicon ultra 0.5 ml 3 kDa cut-off centrifugal filters (Millipore, Ireland). For that, the centrifugal filters were wet with 300 µl of PBS pH 7.0 before adding 200 µl of the eluted protein. After 30 min of centrifugation at 14,000 rpm at 4°C, the volume of the protein was reduced to approximately 100 µl and 400 µl of PBS pH 7.0 were added. This process was repeated four times and the resultant sample collected and stored at −20°C.

### Western blot

Protein expression was confirmed by western blot. IVTT products were diluted 1∶1 in 2x reducing sample buffer (125 mM Tris pH 6.8, 4% SDS, 10% glycerol, 0.006% bromophenol blue, 2% beta-mercaptoethanol) and denatured at 95°C for 5 mins. Samples (2.5 µl of *E. coli* IVTT whole extract or supernatant, and 5 µl of insect cell IVTT whole extract or supernatant) were run in parallel with molecular weight standard (BenchMark pre-stained protein marker, Invitrogen) on a 4–12% NuPage SDS Page gel (Invitrogen, Carlsbad, CA, USA) at 120 V for 70 min. Samples were transferred onto a PVDF membrane (Bio-Rad, Hercules, CA) pre-wet with 100% methanol, using the BioRad Mini Trans-Blot system at 100 V for 1 hr, according to manufacturer's instructions. Membranes were blocked with PBS containing 5% skim milk powder overnight at 4°C with shaking, washed 3 times with PBS containing 0.05% Tween20, then probed with anti-HIS HRP (1∶5000; Roche Diagnostics) or anti-HA HRP (1∶500) (Roche Diagnostics) for 1 hr at room temperature. Membranes were washed 3 times with PBS containing 0.05% Tween before developing with the Immun-Star™ HRP system (Bio-Rad) according to manufacturer's instructions. Exposure times for the X-ray film (FujiFilm Corporation, Tokyo) varied from 5 sec to 4 min. Immunoblot images were acquired in TIF format and analysed using the AnalySIS LS software (version 5.0; Soft Imaging Systems GmbH, Germany).

### Immunization of mice using the VR2516 PyCSP DNA vaccine and PyCSP IVTT product

Mice (n = 5/group) were immunized intramuscularly in the tibialis anterior muscle with 50 µg of VR2516 PyCSP DNA vaccine or VR1020 negative control plasmid, two times at 3 week intervals. Splenocytes were harvested from DNA immunized mice or naïve control mice (n = 5) at 3 weeks post-boost for *in vitro* T cell assays using IVTT products. Serum was collected from select groups for antibody assays. The potential toxicity of IVTT products was assessed prior to use for *in vitro* stimulation of T cell responses by culturing splenocytes (5×10^5^ cells/well) of naïve BALB/c mice with IVTT-produced *Py*CSP either unpurified or purified using NI-NTA resin (Qiagen) or MagneHis particles (Promega) at dilutions of 1∶100, 1∶1000 and 1∶10000, or media alone, and monitoring cell growth for 48 hours. No toxicity due to the unpurified or purified IVTT preparations was apparent, as indicated by cell death.

For the *in vivo* evaluation of IVTT products, mice (*n* = 5/group) were immunized intramuscularly with 50 µg/100 µl of VR2516 *Py*CSP DNA vaccine (split between two legs) and boosted 3 weeks later with *Py*CSP IVTT products formulated with 100 µl Aluminium Hydroxide gel adjuvant (Brenntag Biosector, Frederikssund, Denmark) as follows: A) 15 µl of IVTT reaction (9 µg *Py*CSP/mouse); B) 15 µl of IVTT reaction purified through NI-NTA resin (6.6 µg *Py*CSP/mouse); C) 15 µl of IVTT reaction pu*ri*fied through MagneHis Protein Purification System (3 µg *Py*CSP/mouse); D) 15 µl of IVTT reaction absorbed in 10 µl of Polybead® Poly(methyl methacrylate) microspheres (PMMA microspheres, 0.08–0.09 µm diameter) (Cat #23570, Polysciences, Inc., Warrington, PA, USA) (9 µg *Py*CSP/mouse); and E) 15 µl of IVTT reaction associated to 10 µl of Protein G conjugated microspheres/carboxylate beads (Cat #21106, Polyscience) (9 µg *Py*CSP/mouse). The beads used in the immunizations were washed 3 times with PBS pH 7.0 before addition of the IVTT product, and used without further processing. Positive and negative controls groups (*n* = 5) were administered 50 µg of VR2516 *Py*CSP DNA vaccine intramuscularly or 100 µl of Alum mixed with 100 µl of PBS pH 7.0, respectively. Three weeks after immunization mice were sacrificed and the spleens harvested for use as effectors in T cell assays.

### *In vitro* culture of murine splenocytes

Spleens were macerated, washed in Dulbecco's solution containing 2% FCS, the red blood cells lysed with 0.09% NH_4_Cl for 5 min at 37°C, and then washed again. The splenocytes were resuspended in complete DMEM containing 10% FCS, 100 U/ml of Penicillin, 100 µg/ml of Streptomycin, 2 mM L-Glutamine and 0.05 mM ß-mercaptoethanol, and cultured in 96-wells flat-bottomed plates for ELIspot or CBA at a concentration of 5×10^5^ cells/well.

For *in vitro* evaluation of IVTT products, splenocytes were stimulated with unpurified r*Py*CSP IVTT; r*Py*CSP IVTT associated to Polybeads or ProteinG beads; or r*Py*CSP IVTT purified using NI-NTA resin or MagneHis Ni-particles. Unpurified IVTT products were used at a dilution of 1∶200 or 1∶1000 and purified IVTT products were used at a dilution of 1∶100 or 1∶200 for evaluation of cytokines in the supernatant using the Cytometric Bead Array assay or IFN-γ secreting cells via ELIspot, respectively. Pilot experiments were carried out to find out the optimal dilutions of IVTT products for these assays (data not shown). An IVTT reaction using the empty pIVEX HisHA vector was used as negative control. A pool of synthetic peptides representing defined CD4^+^ and CD8^+^ T cell epitopes from *Py*CSP (residues 57–70, sequence KIYNRNIVNRLLGD [52]; residues 280–288, sequence SYVPSAEQI [53]; and resides 280–295, sequence SYVPSAEQILEFVKQI [54]) at 10 µg/ml of each peptide was used as a positive control. ConA (Sigma-Aldrich, St. Louis, MO) at 5 µg/ml was used as a mitogen control. All stimuli were added in association with 1×10^5^ cells/well of A20/2 J cells (ATCC clone HB-98) irradiated at 1600 rads, as antigen presenting cells.

For *in vivo* evaluation of IVTT products, splenocytes were stimulated with 2×10^4^ cells/well of A20 cells transfected with VR2516 *Py*CSP DNA or VR1020 negative control using the AMAXA Nucleofector transfection kit (AMAXA, Cologne, Germany) according to manufacturer's manual, or with 1×10^5^ cells/well of A20 cells pulsed with the PyCSP peptide pool. A20 cells alone and medium were used as negative controls and ConA used as mitogen control. AMAXA transfections included parallel reactions with GFP plasmid as a positive control (data not presented); additionally, our laboratory routinely uses AMAXA-transfected A20 cells as APCs for in vitro T cell assays [55], [56].

### *In vitro* culture of human PBMCs

Peripheral blood mononuclear cells (PBMC) were isolated using standard Ficoll density gradient centrifugation and resupsended in complete RPMI containing 10% human AB serum, 100 units/ml of Penicillin, 100 µg/ml of Streptomycin, 2 mM L-Glutamine, 1 mM Sodium Pyruvate and 25 mM Hepes. Cells were cultured at a concentration of 1×10^5^ cells/well in 96-wells round-bottomed plates at 37°C in an atmosphere of 5% CO_2_. The cells were stimulated with IVTT-produced FluM, FluHA, CMVpp65 and EBNA3A antigens, either unpurified; associated to Polybeads or ProteinG beads; or purified using NI-NTA resin or MagneHis Ni-particles. Unpurified and purified IVTT products were used at a dilution of 1∶100, 1∶1000 or 1∶10000 for evaluation of cytokines in the supernatant using the Cytometric Bead Array assay, or at a dilution of 1∶1000 (unpurified) or 1∶100 (purified) for evaluation of IFN-γ secreting cells via ELISpot. Negative controls included empty pIVEX HisHA IVTT reaction alone or associated to Polybeads or ProteinG beads, or medium only. A CEF peptide pool consisting of 32 synthetic peptides representing defined CD8^+^ T cell epitopes from human Cytomegalovirus, Epstein-Barr Virus and Influenza Virus (Anaspec, San Jose, CA) at 5 µg/ml total was used as a positive control [44]. Phytohemagglutinin (PHA; Sigma-Aldrich, St. Louis, MO) at 2 µg/ml was used as a mitogen control.

### IFN-γ ELIspot assay

IFN-γ secreting T cells were enumerated by ELISpot. Briefly, MultiScreen HTS IP 96 plates (Cat MSIPS4510, Millipore, Ireland) were pre-wet with 15 µl/well of 35% ethanol and washed twice with PBS (pH 7.4). For mouse ELIspot assays, wells were coated with 75 µl/well of sterile PBS (pH 7.4) containing 10 µg/ml anti-mouse IFN-γ (Cat 551216, Clone R4-6A2, BD Pharmingen, CA) with or without 1∶500 anti-His mAb (Cat H1029, Sigma-Aldrich, St. Louis, MO) overnight at room temperature. For human ELIspot assays, wells were coated with 75 µl/well of carbonate buffer (pH 9.6) containing 10 µg/ml anti-human IFN-γ (Cat 3420-3, Clone 1-D1K, MABTECH, Sweden) with or without 1∶500 anti-His mAb (Cat H1029, Sigma-Aldrich, St. Louis, MO) overnight at 4°C. Plates were washed twice with 200 µl/well DMEM (splenocytes) or RPMI (PBMC) and blocked with 200 µl/well of medium containing 10% FCS for 3 hrs at 37°C. After blocking, the wells coated with anti-His mAb were incubated with *Py*CSP, FluM, FluHA, CMVpp65, EBNA3A or empty pIVEX HisHA IVTT products diluted 1∶1000 in DMEM or RPMI containing 2% FCS in a volume of 50 µl/well, overnight at 4°C (to absorb the His-tagged IVTT products to the anti-His mAb coated wells). Plates were washed 3 times with DMEM or RPMI, and the cells added to the IFN-γ and His coated wells in triplicates. Alternatively, in the absence of anti-His pre-coating, IVTT products unpurified, associated to Polybeads or ProteinG beads, or purified using NI-NTA resin or MagneHis Ni-particles (see above), were added to the IFN-γ coated wells together with the cells (100,000 PBMCs or 500,000 splenocytes), in triplicate. After 36 hrs incubation, plates were flicked to remove the cells and washed 6 times with PBS-Tween 0.05% pH 7.4 (PBS-T). Then 75 µl/well of biotinylated anti-mouse IFN-γ (Cat 554410, Clone XMG1.2, BD Pharmingen) at 2 µg/ml in PBS or 75 µl of biotinylated anti-human IFN-γ (Cat 3420-6, Clone 7-B6-1, MABTECH, Sweden) diluted 1∶1000 in PBS was added to each well. Plates were incubated for 3 hrs at room temperature, washed 3 times with PBS-T, and 75 µl/well of Streptavidin-HRP for splenocytes cultures or Streptavidin-AP (Bio-Rad, Hercules, CA) for PBMC cultures diluted 1∶1000 in PBS was added. After 1 hour incubation at room temperature, plates were washed 3 times with PBS-T followed by 3 times with PBS alone, and developed using the AEC substrate set (BD Pharmingen) for mouse splenocytes or SigmaFast BCIP/NBT (Sigma-Aldrich) for humans PBMC cultures, according to manufacturer's instructions. After 10 mins, the plates were rinsed extensively with dH2O to stop the enzymatic reaction, dried and stored in the dark. The number of IFN-γ secreting cells was determined using the automated ELISpot reader (AID iSpot Reader, Autoimmun Diagnostika GmbH, Strassberg, Germany).

### Cytometric Bead Array assay

To determine the cytokines secreted by the stimulated splenocytes or PBMC, cells were cultured at a concentration of 5×10^5^ cells/well in 96-wells flat-bottomed plates (Corning Incorporated, Corning, NY, USA). The supernatant was collected after 48 hrs and 72 hrs stimulation and analyzed with Cytometric Beads Array flex kits (BD Biosciences, San Jose, CA, USA) containing IL-1ß, IL-2, IL-4, IL-5, IL-6, IL-10, IL-12p70, IL-13, IFN-γ and TNF-α for mouse and IL-10, IFN-γ and TNF-α for human samples. Data were acquired and analyzed using BD FACS Array Bioanalyser and FlowJo 7.6 software.

### ELISA

*Py*CSP-specific antibody responses in mouse sera were evaluated by peptide ELISA, as previously described [57]. Briefly, flat-bottomed 96-well microtiter plates (Immulon 4; Dynex Technology Inc., Chantilly, VA, USA) were coated with 100 µl/well of recombinant PyCSP protein at a concentration of 1 µg/ml in carbonate-bicarbonate coating buffer pH 7.4, and incubated overnight at 4°C. Wells were blocked for 1 hr with 2% BSA in PBS containing 0.05% Tween 20 (Blocking Buffer) and washed three times with PBS containing 0.05% Tween 20 (PBS-T). Consecutive dilutions of individual sera diluted in PBS containing 0.01% Tween-20 were incubated for 2 hrs at room temperature. The plates were washed 3 times, and incubated with 100 µl/well Biotinylated anti-mouse IgG (Jackson ImmunoResearch) at a dilution of 1∶20,000 for 1 hr. The plates were washed three times and incubated with Streptavidin HRP (BD Biosciences) at a dilution of 1∶1000 for 1 hr. The plates were washed and developed for 10 mins with 50 µl/well TMB substrate (Sigma). Reactions were terminated by adding 50 µl of stopping buffer and the OD450 recorded using a VersaMax microplate reader (Molecular Devices, Sunnyvale, CA, USA). Results are expressed as mean OD readings of triplicate wells +/- SE.

### Statistical analysis

Data from the *in vitro* and *in vivo* tests in mice were analyzed by the Student *t*-test and two-way ANOVA, respectively, where a *p* value<0.05 was considered significant. Data from human PBMC ELIspot and CBA were analyzed using two-way ANOVA and one-way ANOVA, respectively. A *p* value<0.05 was considered significant.

## Supporting Information

Figure S1 **Recombinants produced using** ***E. coli*** **cell-free IVTT system.** Western Blot of (A) viral antigens FluM (32 kDa), FluHA (68 kDa), CMVpp65 (68 kDa) and EBNA3A (108 kDa), and parasite protein PyCSP (44 kDa), probed with anti-HA antibody, and (B) viral antigens FluM, FluHA, CMVpp65 and EBNA3A probed with anti-His antibody. Whole IVTT extracts (5 µl) of each antigen were run on a 12% NUPAGE gel, transferred to a PVDF membrane, and probed with anti-HA HRP antibody (1∶500 dilution) or anti-His HRP antibody (1∶5000 dilution). The western probed with anti-HA also detected a cross reactive band of 56 kDa in all expression extracts. The western probed with anti-His mAb showed the presence of partial products probably due to early termination of translation.
(TIF)

Figure S2 **Antigen-specific TNF-α and IL-10 responses of mouse splenocytes stimulated** ***in vivo*** **or** ***in vitro*** **with IVTT-proteins.** (A) and (B): splenocytes of mice immunized with VR2516 *Py*CSP plasmid DNA or VR1020 control DNA were cultured *in vitro* with unpurified r*Py*CSP IVTT; r*Py*CSP IVTT associated to Polybeads or ProteinG beads; r*Py*CSP IVTT purified using NI-NTA resin, MagneHis Ni-particles, or anti-HIS; or synthetic peptides representing defined T cell epitopes from PyCSP (positive control), as indicated. (C) and (D): splenocytes of mice immunized with VR2516 *Py*CSP plasmid DNA and boosted *in vivo* with unpurified r*Py*CSP IVTT; r*Py*CSP IVTT associated to Polybeads or ProteinG beads; or r*Py*CSP IVTT purified using NI-NTA resin or MagneHis Ni-particles; all formulated with Alum adjuvant. Parallel groups of mice were boosted with either Alum only or VR2516 as controls. Splenocytes were cultured *in vitro* with A20 cells transfected with VR2516 *Py*CSP plasmid DNA or A20 cells pulsed with synthetic peptides representing defined *Py*CSP T cell epitopes, as indicated. Secreted TNF-α (A and C) or IL-10 (B and D) in culture supernatant was measured by Cytometric Bead Array (CBA) after 48 hrs stimulation. *P<0.05, **P<0.01 and ***P<0.001 compared to negative controls.
(TIF)

Figure S3 **Antigen-specific TNF-α responses by human PBMC stimulated with IVTT-proteins.** PBMCs were cultured with IVTT-produced FluM, FluHA, CMVpp65 and EBNA3A purified using (A) NI-NTA nickel resin or (B) MagneHis Ni-particles; (C) unpurified; associated to (D) ProteinG beads or (E) Polybeads; or (F) added to wells precoated with Anti-His; IVTT products were diluted 1∶100, 1∶1000, or 1∶10,000. Secreted TNF-α in the supernatant of cultured PBMCs was analyzed by Cytometric Bead Array after 48 hrs stimulation. * *P*<0.05 compared to negative controls.
(TIF)

Figure S4 **Antigen-specific IL-10 responses by human PBMC stimulated with IVTT-proteins.** PBMCs were cultured with IVTT-produced FluM, FluHA, CMVpp65 and EBNA3A purified using (A) NI-NTA nickel resin or (B) MagneHis Ni-particles; (C) unpurified; associated to (D) ProteinG beads or (E) Polybeads; or (F) added to wells pre-coated with Anti-His; IVTT products were diluted 1∶100, 1∶1000, or 1∶10,000. Secreted IL-10 in the supernatant of cultured PBMCs was analyzed by Cytometric Bead Array after 48 hrs stimulation. * *P*<0.05 compared to negative controls.
(TIF)
