# Supplementary figures and images for: Evaluation of Approaches to Identify the Targets of Cellular Immunity on a Proteome-Wide Scale
Source: PLoS One. 2011 Nov 11;6(11):e27666. doi: 10.1371/journal.pone.0027666 (PMC3214079; doi:10.1371/journal.pone.0027666)

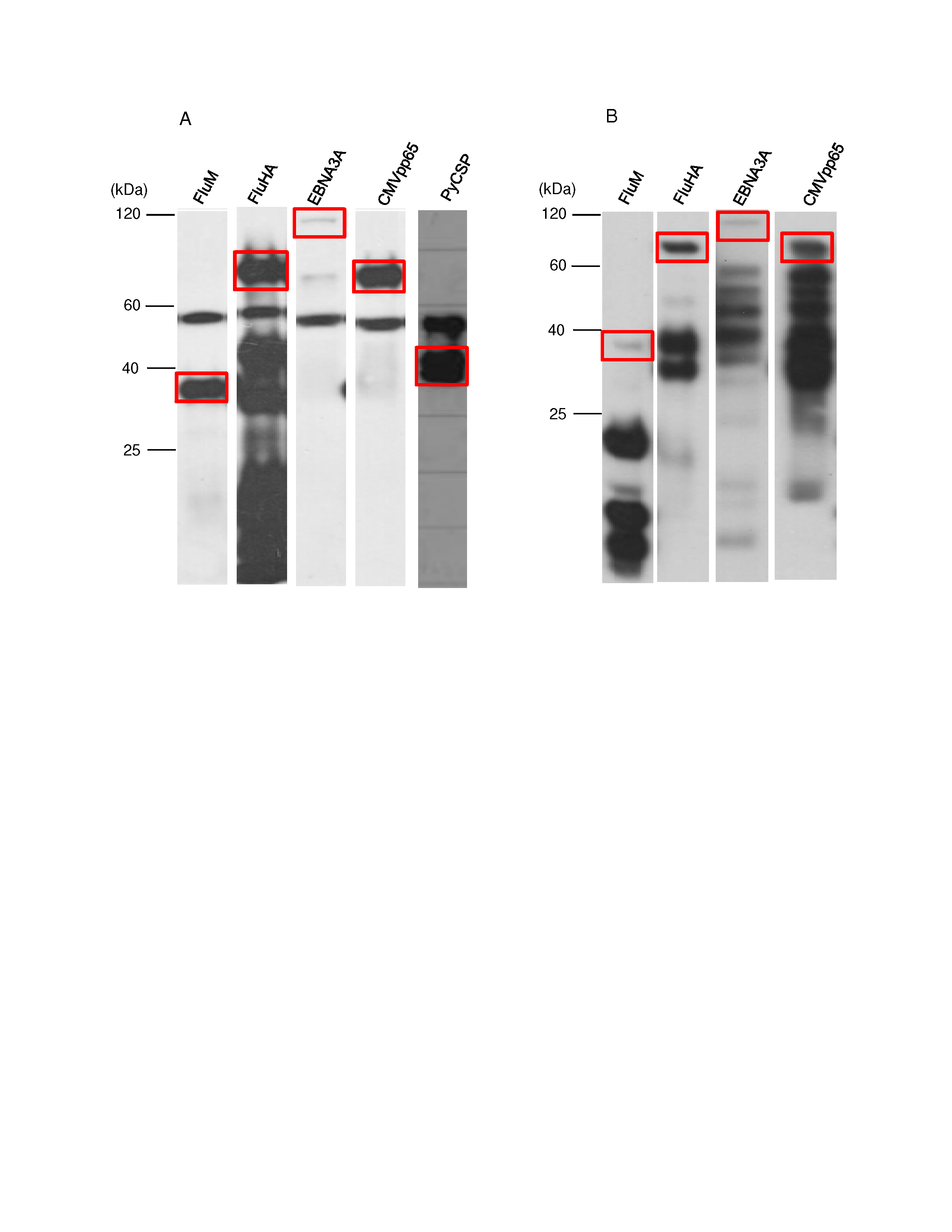

Supplement: Figure S1 — Recombinants produced using E. coli cell-free IVTT system. Western Blot of (A) viral antigens FluM (32 kDa), FluHA (68 kDa), CMVpp65 (68 kDa) and EBNA3A (108 kDa), and parasite protein PyCSP (44 kDa), probed with anti-HA antibody, and (B) viral antigens FluM, FluHA, CMVpp65 and EBNA3A probed with anti-His antibody. Whole IVTT extracts (5 µl) of each antigen were run on a 12% NUPAGE gel, transferred to a PVDF membrane, and probed with anti-HA HRP antibody (1∶500 dilution) or anti-His HRP antibody (1∶5000 dilution). The western probed with anti-HA also detected a cross reactive band of 56 kDa in all expression extracts. The western probed with anti-His mAb showed the presence of partial products probably due to early termination of translation. (TIF) [file pone.0027666.s001.tif]

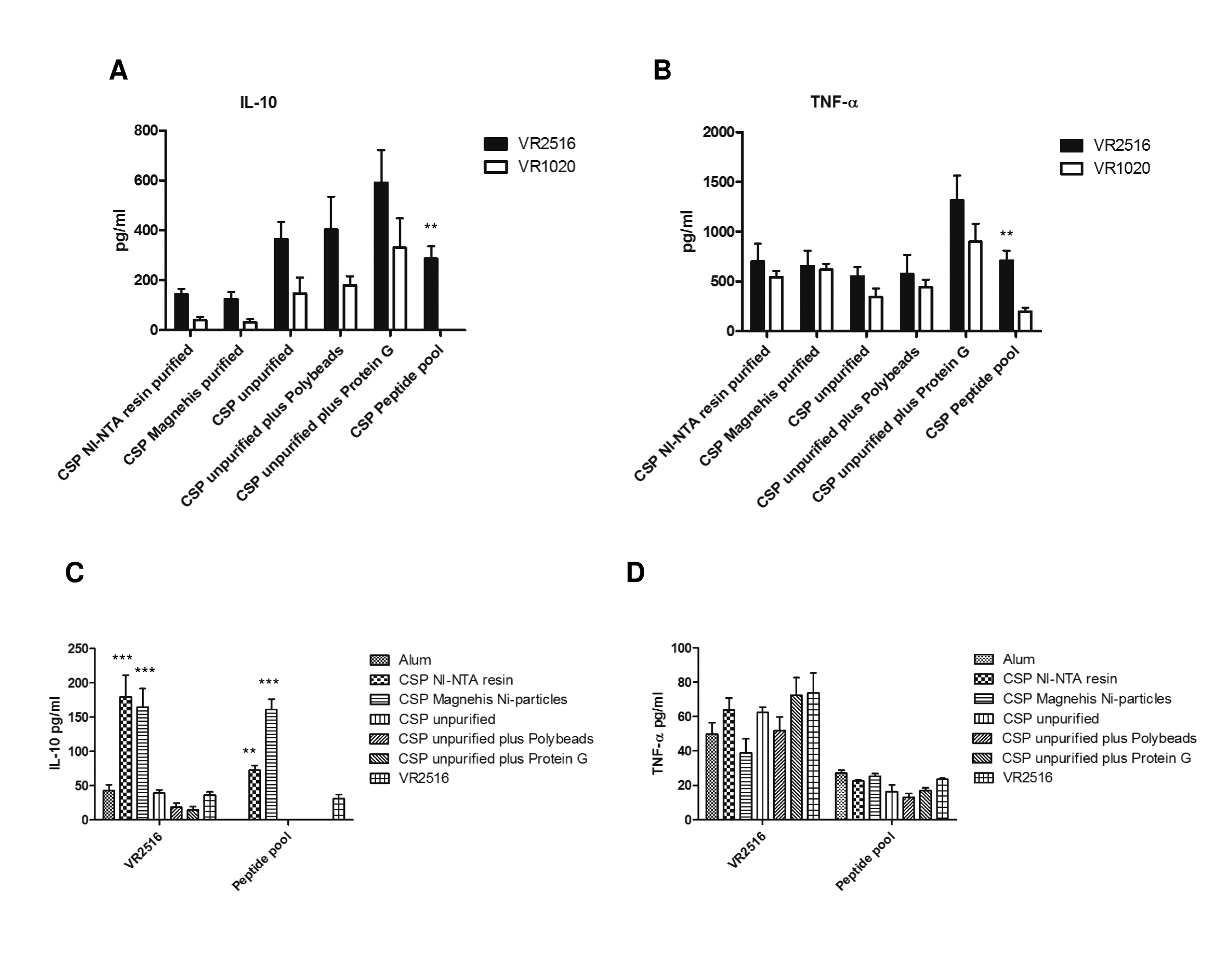

Supplement: Figure S2 — Antigen-specific TNF-α and IL-10 responses of mouse splenocytes stimulated in vivo or in vitro with IVTT-proteins. (A) and (B): splenocytes of mice immunized with VR2516 PyCSP plasmid DNA or VR1020 control DNA were cultured in vitro with unpurified rPyCSP IVTT; rPyCSP IVTT associated to Polybeads or ProteinG beads; rPyCSP IVTT purified using NI-NTA resin, MagneHis Ni-particles, or anti-HIS; or synthetic peptides representing defined T cell epitopes from PyCSP (positive control), as indicated. (C) and (D): splenocytes of mice immunized with VR2516 PyCSP plasmid DNA and boosted in vivo with unpurified rPyCSP IVTT; rPyCSP IVTT associated to Polybeads or ProteinG beads; or rPyCSP IVTT purified using NI-NTA resin or MagneHis Ni-particles; all formulated with Alum adjuvant. Parallel groups of mice were boosted with either Alum only or VR2516 as controls. Splenocytes were cultured in vitro with A20 cells transfected with VR2516 PyCSP plasmid DNA or A20 cells pulsed with synthetic peptides representing defined PyCSP T cell epitopes, as indicated. Secreted TNF-α (A and C) or IL-10 (B and D) in culture supernatant was measured by Cytometric Bead Array (CBA) after 48 hrs stimulation. *P<0.05, **P<0.01 and ***P<0.001 compared to negative controls. (TIF) [file pone.0027666.s002.tif]

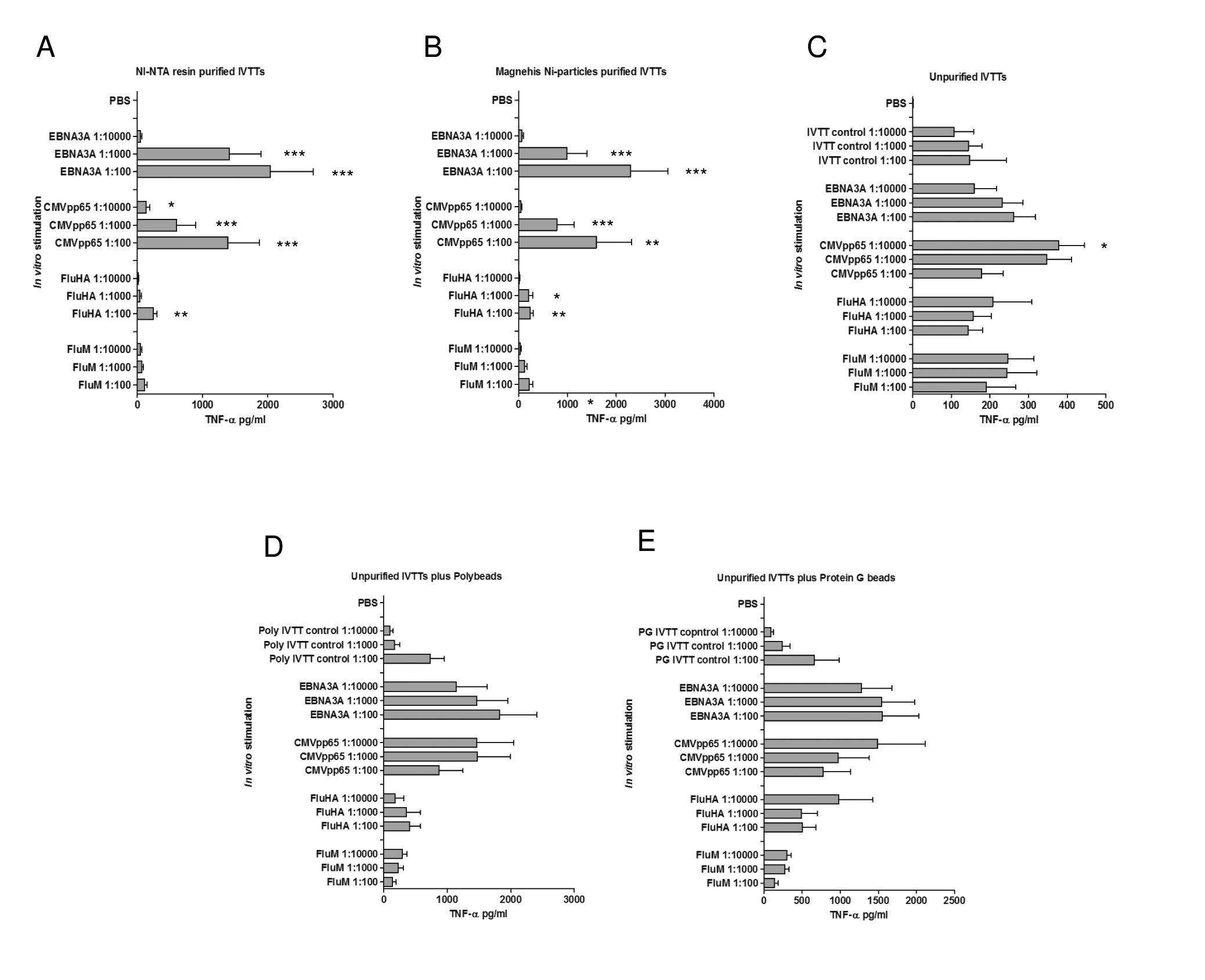

Supplement: Figure S3 — Antigen-specific TNF-α responses by human PBMC stimulated with IVTT-proteins. PBMCs were cultured with IVTT-produced FluM, FluHA, CMVpp65 and EBNA3A purified using (A) NI-NTA nickel resin or (B) MagneHis Ni-particles; (C) unpurified; associated to (D) ProteinG beads or (E) Polybeads; or (F) added to wells precoated with Anti-His; IVTT products were diluted 1∶100, 1∶1000, or 1∶10,000. Secreted TNF-α in the supernatant of cultured PBMCs was analyzed by Cytometric Bead Array after 48 hrs stimulation. * P<0.05 compared to negative controls. (TIF) [file pone.0027666.s003.tif]

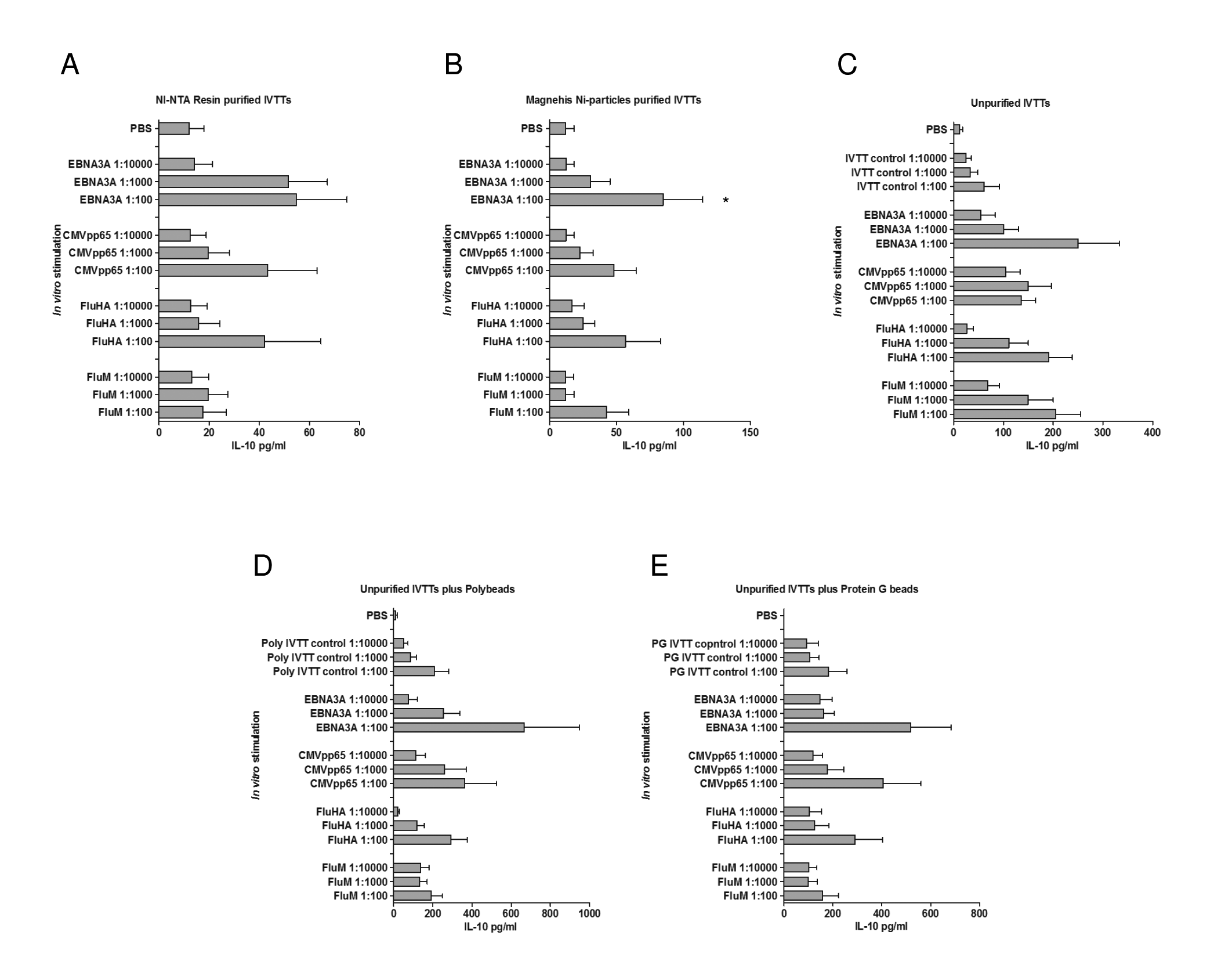

Supplement: Figure S4 — Antigen-specific IL-10 responses by human PBMC stimulated with IVTT-proteins. PBMCs were cultured with IVTT-produced FluM, FluHA, CMVpp65 and EBNA3A purified using (A) NI-NTA nickel resin or (B) MagneHis Ni-particles; (C) unpurified; associated to (D) ProteinG beads or (E) Polybeads; or (F) added to wells pre-coated with Anti-His; IVTT products were diluted 1∶100, 1∶1000, or 1∶10,000. Secreted IL-10 in the supernatant of cultured PBMCs was analyzed by Cytometric Bead Array after 48 hrs stimulation. * P<0.05 compared to negative controls. (TIF) [file pone.0027666.s004.tif]
